# Common reduction of the Raf kinase inhibitory protein in clear cell renal cell carcinoma

**DOI:** 10.18632/oncotarget.1558

**Published:** 2014-04-24

**Authors:** Brianne Hill, Jason De Melo, Judy Yan, Anil Kapoor, Lizhi He, Jean-Claude Cutz, Xingchang Feng, Nazihah Bakhtyar, Damu Tang

**Affiliations:** ^1^ Division of Nephrology, Department of Medicine, McMaster University, Hamilton, Ontario, Canada, Hamilton, Ontario, Canada; ^2^ Father Sean O'Sullivan Research Institute, Hamilton, Ontario, Canada; ^3^ The Hamilton Center for Kidney Research, St. Joseph's Hospital, Hamilton, Ontario, Canada; ^4^ Division of Urology, Department of Surgery, McMaster University, Hamilton, Ontario, Canada; ^5^ Department of Biological Chemistry and Molecular Pharmacology (BCMP), Harvard Medical School, Boston, MA, USA; ^6^ Department of Pathology, McMaster University, Hamilton, Ontario, Canada; ^7^ College of Veterinary Medicine, Northeast Agricultural University, Harbin, China 150030

**Keywords:** RKIP, Raf, ERK, ccRCC, and kidney cancer

## Abstract

Despite the recent progress in our understanding of clear cell renal cell carcinomas (ccRCCs), the etiology of ccRCC remains unclear. We reported here a prevailing reduction of the raf kinase inhibitory protein (RKIP) in ccRCC. In our examination of more than 600 ccRCC patients by western blot and immunohistochemistry, *RKIP* was significantly reduced in 80% of tumors. Inhibition of RKIP transcription in ccRCC occurs to greater levels than *VHL* transcription based on the quantification analysis of their transcripts in six large datasets of DNA microarray available in Oncomine™ with the median rank of suppression being 582 and 2343 for RKIP and VHL, respectively. Collectively, the magnitude of RKIP reduction and the levels of its downregulation match those of VHL. Furthermore, RKIP displays tumor suppressing activity in ccRCC. While modulation of RKIP expression did not affect the proliferation of A498 and 786-0 ccRCC cells and neither their ability to form xenograft tumors in NOD/SCID mice, ectopic expression or knockdown of RKIP inhibited or enhanced A498 and 786-0 ccRCC cell invasion, respectively. This was associated with robust changes in vimentin expression, a marker of EMT. Taken together, we demonstrate here that downregulation of RKIP occurs frequently at a rate that reaches that of VHL, suggesting RKIP being a critical tumor suppressor for ccRCC. This is consistent with RKIP being a tumor suppressor for other cancers.

## INTRODUCTION

Renal cell carcinoma (RCC) accounts for 85% of kidney cancer cases. In 2008, 54,390 new cases of RCC were diagnosed in the United States and 13,010 (23.9%) of the patients died from the disease [1]. The main histological types of RCC are clear cell (ccRCC, 75%), papillary (pRCC, 12%), chromophobe (cRCC, 4%), and oncocytoma (4%) [2]. Clear cell RCC is the most prevalent, the most aggressive [3], and the most common cause of kidney cancer-associated deaths [4].

Loss of VHL is a critical event for ccRCC tumorigenesis. Patients with germline mutations of *VHL* are at risk of developing ccRCC with up to 600 tumors and 1100 cysts per kidney [5]; somatic mutations leading to biallellic inactivation of *VHL* occur in 50%–60% of sporadic ccRCCs [6]; *VHL* promoter methylation was observed in 15% of ccRCCs [7]. A recent report revealed an even higher rate (82.4%) of *VHL* somatic mutations and 8.3% of the *VHL* promoter CpG island hypermethylation [8]. Collectively accumulating evidence clearly demonstrates VHL being a critical tumor suppressor of ccRCC. However, loss of VHL is not sufficient. Patients with *VHL* deficiency develop ccRCCs that are often preceded by pre-neoplastic renal cysts [9] and mice deficient in *VHL* in the proximal tubule epithelium only develop low levels of renal cysts [10], demonstrating the requirement of other oncogenic events.

RKIP suppresses multiple oncogenic pathways [11,12]. The protein binds to the N-terminus of Raf-1, preventing its interaction with MEK, an event that is required for MEK activation [13,14,11]; the association also impedes phosphorylation of Raf-1 at serine 338 (S338) and tyrosine 341 (Y341) [15], mandatory events for Raf-1 activation [16,17]. Furthermore, growth factor signals which activate Raf-1/MEK/ERK pathway induce phosphorylation of RKIP at S153, resulting in RKIP being incapable of binding Raf-1 [18]. RKIP indirectly inhibits Wnt signaling by binding and stabilizing the glycogen synthase kinase 3-β (GSK3β) [19], and RKIP impedes NF-κB function by down-regulation of IκB kinase activity [20]. NF-κB plays a major role in up-regulation of Snail [21,22]. Snail is a major contributor of EMT, an essential process of cancer metastasis [23,24]. Consistent with these observations, the polycomb protein EZH2 was reported to promote the invasion of breast and prostate cancers via suppression of RKIP transcription [25], and RKIP has been demonstrated to inhibit metastasis of multiple human cancers, including prostate, breast, melanoma, and colorectal cancers, and gastrointestinal stromal tumors [26-32]. However, it remains unclear whether RKIP is also a tumor suppressor of ccRCC.

By using 2D gel-coupled mass spectrometry, western blot, and immunohistochemistry (IHC), we observed a common RKIP reduction in 80% of more than 600 cases of ccRCC examined. The levels of RKIP reduction follow ccRCC progression and metastasis. Furthermore, modulation of RKIP in ccRCC A498 and 786-0 cells accordingly affected vimentin expression, a marker of EMT, along with affecting ccRCC cell invasion. Collectively, we provide evidence that RKIP can be an important tumor suppressor of ccRCC.

## RESULTS

### Common reduction of RKIP in ccRCC

We have collected RCC and the adjacent non-tumor kidney (ANK) tissue from 90 RCC patients. Tissue lysates were analyzed for actin by western blot and patients where actin was not readily detected in either tissue (data not shown) were excluded. The resulting patient cohort consisted of 50 ccRCC (Supplementary Table 1), 13 pRCC, and other histological types of RCC (data not shown). We subsequently focused on ccRCC, as the number of cases for the other histological types was not sufficient for a valid statistical analysis.

**Figure 1:**
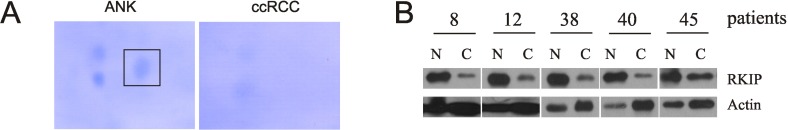

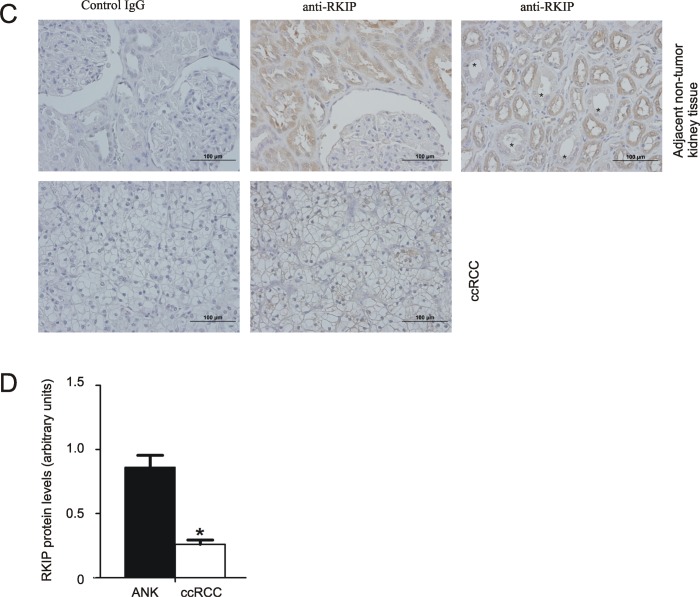
Reduction of RKIP in ccRCC **(A)** ccRCC and its adjacent non-tumor kidney tissue lysate was separated using 2-D gel (pH 3-10). Signals were developed by coomassie blue staining. The unique spot in the adjacent non-tumor kidney tissue was indicated. **(B)** Western blot analysis for RKIP in ccRCC and ANK for 50 ccRCC patients. Typical results for the indicated patients are shown. **(C)** Immunohisochemistry (IHC) staining for RKIP in ANK and ccRCC. The asterisks indicate kidney distal tubules. **(D)** Based on western blot analysis of RKIP in ANK and ccRCC tissues for 50 ccRCC patients (Supplementary Table 1), RKIP in ANK and ccRCC was normalized to the respective actin (Supplementary Table 1). Means ± SE (standard error) of the RKIP protein in ANK and ccRCC are graphed. * *p* ≤ 0.05 (2-tailed student t-test).

To find unique proteins present in either ccRCC or ANK tissues, we performed 2D-gel analysis for several patients. One spot with a molecular weight of approximately 20 kDa and pI 7.42 was commonly detected in ANK, which was consistently reduced in the respective ccRCC (Fig. 1A). Mass spectrometry analysis of the spot recovered 97.3% of peptide sequences, which matched to the mammalian RKIP (data not shown). To confirm RKIP reduction, RKIP in ccRCC and ANK tissues was examined by western blot and IHC. In comparison to ANKs, dramatic reductions of RKIP in ccRCC were clearly detected (Fig. 1B, C). Additionally, RKIP was primarily expressed in the proximal tubules (Fig. 1C). As ccRCC is widely regarded to derive from the proximal tubular epithelial cells [9,33,34], the reduction of RKIP in ccRCC suggests RKIP being a candidate tumor suppressor of ccRCC. This concept is consistent with the reports that RKIP is a tumor suppressor for a variety of human cancers [11,12].

**Figure 2:**
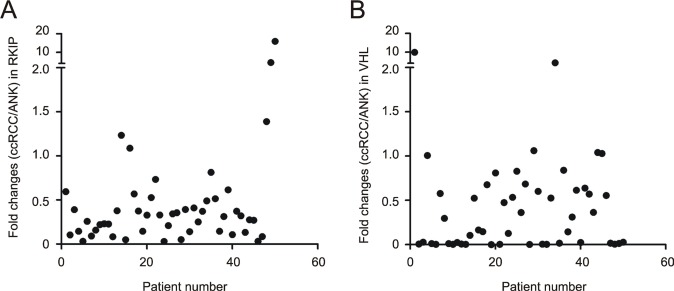
The magnitude of reduction of RKIP and VHL in ccRCC Levels of RKIP and VHL in ccRCC and the ANK tissues for 50 ccRCC patients were determined by western blot, followed by normalization to the respective actin. The ratios of RKIP **(A)** and VHL **(B)** in ccRCC versus ANK (ccRCC/ANK) were determined and plotted.

To quantify RKIP, we analyzed RKIP in 50 ccRCC and the respective ANK tissues. After normalization to actin, the RKIP protein levels in ccRCC were significantly lower than those in the ANK tissues (Fig. 1D). Except for 5 patients, the ratios of RKIP protein in ccRCC versus ANK (ccRCC/ANK) were below 1.0 in 90% (45/50) of ccRCC (Fig. 2A, Supplementary Table 1). Since the typical feature of ccRCC is the common reduction of VHL in up to 91% of ccRCC, we also examined the VHL protein. In 50 patients, 88% (44/50) of ccRCCs displayed reduced VHL in comparison to ANKs (Fig. 2B). To further investigate RKIP downregulation in comparison to VHL reduction, we determined their expressions in 6 datasets of DNA microarray (Oncomine™) [35-40]. *RKIP* transcription was suppressed to greater levels than that of *VHL* in all 6 datasets with the median rank of suppression being 582 and 2343 for RKIP and VHL, respectively (Table 1). Taken together, reduction of RKIP in ccRCC reaches the magnitude and the level of VHL downregulation (Fig. 2). As 20 ccRCCs showed undetectable VHL (Fig. 2B, see the dots on the X-axis) versus 5 ccRCC with undetectable RKIP (Fig. 2A), the tumorigenesis of ccRCC most likely relies heavily on the loss of VHL function and on the partial reduction of RKIP action.

### Reduction of RKIP generally correlates with ccRCC progression

**Figure 3:**
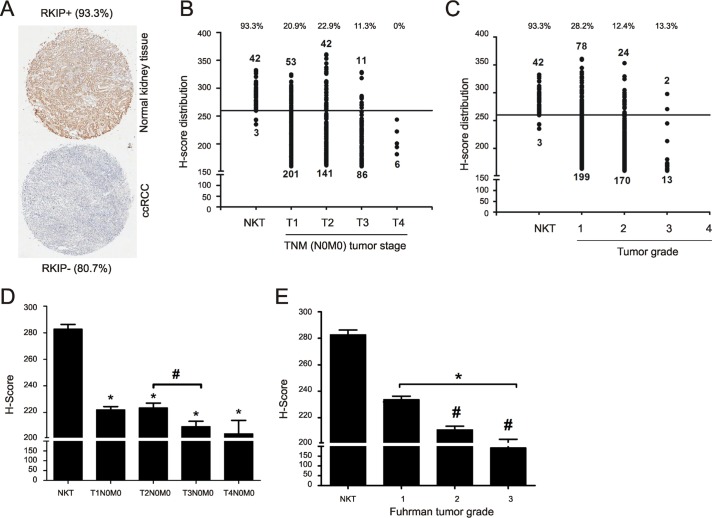

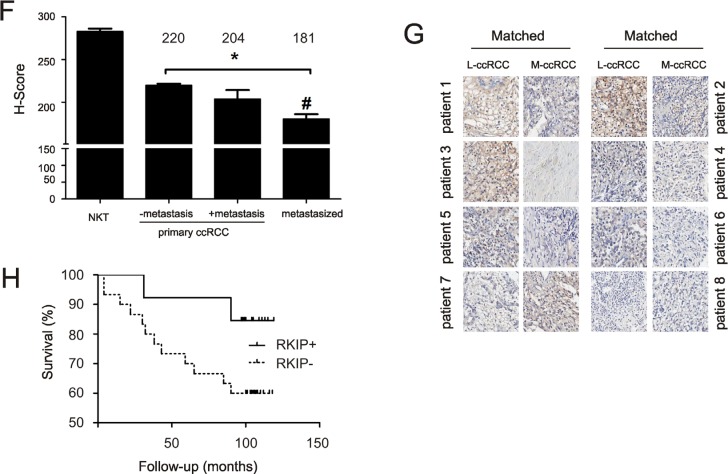
Reduction of RKIP correlates with ccRCC progression **(A)** Typical images of RKIP staining for normal kidney and ccRCC tissues in a set of TMA containing 540 patients with organ-confined ccRCC. The percentage of tissues with strong (normal kidney tissue) or weak (ccRCCs) RKIP staining are indicated. (**B and C**) Numbers of tissues with strong (H-Score ≥ 260) versus weak staining (H-Score < 260) of RKIP are graphed based on the TNM stages and Fuhrman grade as indicated. The percentages of tissues with strong RKIP staining for normal kidney tissues (NKT) and individual tumor groups are indicated (top). (**D and E**) The average of RKIP expression (H-Scores) ± SE for normal kidney tissues and ccRCCs in individual TNM stages (**D**) and Fuhrman grades (**E**) are graphed. * *p* < 0.001 between normal kidney tissues and the ccRCCs of individual TNM stages (**D**) or Fuhrman grades (**E**) (one-way ANOVA); # *p* < 0.05 between T2N0M0 and T3N0M0/T4N0M0 (D); # *p* < 0.001 between Fuhrman grades 1 and 2/3 (**E**) (one-way ANOVA). (**F**) RKIP expression in normal kidney tissue (NKT), primary ccRCC without (-) or with (+) metastasis, and metastasized ccRCC (means ± SE). * *p* < 0.001 between NKT and ccRCCs (one-way ANOVA); # *p* < 0.05 between primary ccRCC with metastasis and metastasized ccRCC (one-way ANOVA). The average H-Scores for individual groups of ccRCC are indicated. (**G**) Representative images of RKIP staining for the matched primary and metastasized ccRCC for 8 patients. (**H**) Kaplan-Meier surviving analysis for patients with ccRCC expressing RKIP at H-Score ≤ 204 (RKIP-, 30 patients) or > 204 (RKIP+, 13 patients). *p* = 0.1141 by Mantel-Cox Test and *p* = 0.1052 by Gehan-Breslow-Wilcoxon Test.

To further investigate the relationship between RKIP reduction and ccRCC tumorigenesis, a set of tissue microarray (TMA) slides (KD806, KD951, KD2085, KD2088 and KD6161) from US Biomax were used, which contained 45 cases of normal kidney tissues (NKT) and 571 ccRCCs (556 organ-confined tumors without metastasis and 15 carcinomas with metastasis). Sixteen cases were excluded because of poor quality. Among 45 NKTs, 93.3% showed readily detectable RKIP (Fig. 3A); among 540 cases of ccRCC, 80.7% displayed dramatically reduced RKIP (Fig. 3A). The percentage of RKIP reduction in these ccRCC populations is consistent with that observed in our patient cohort.

**Table 1:** The expression of RKIP and VHL mRNA in ccRCC

				mRNA Under-expression				
Dataset	Sample Set^1^	Genes^2^	Samples Examined	RKIP	*p*-value	VHL	*p*-value	Reference
Beroukim Renal	Hereditary ccRCC vs. Normal	12,624	70	330	1.34E-8	1395	2.12E-5	Beroukhim et al., 2009
Beroukim Renal	Non-Hereditary ccRCC vs. Normal	12,624	70	582	1.61E-6	2343	0.002	Beroukhim et al., 2009
Gumz Renal	ccRCC vs. Normal	12,624	20	416	2.90E-6	1415	0.075	Gumz et al., 2007
Higgins Renal	ccRCC vs. Normal	5,900	44	424	0.002	1521	3.15E-6	Higgins et al., 2003
Jones Renal	ccRCC vs. Normal	17,779	18	1566	0.002	2972	0.015	Jones et al., 2005
Lenburg Renal	ccRCC vs. Normal	12,624	92	2064	1.49E-4	9030	0.389	Lenburg et al., 2003
Yusenko Renal	ccRCC vs. Normal	19,574	67	5339	0.137	13201	0.757	Yusenko et al., 2009
			**Median Rank**	**582**	**1.61E-6**	**2343**	**0.002**	

Oncomine™ (Compendia Bioscience, Ann Arbor, MI) was used to analyze mRNA microarray data from 6 available data sets. Comparisons are indicated in the Sample set column.

1 Only ccRCC cases in the datasets were used;

2 The number of genes analyzed.

We subsequently examined RKIP expression in the course of ccRCC progression. The organ-confined ccRCCs could be categorized according to either TNM (T: primary tumor size, N: lymph node metastasis, M: long distance metastasis) tumor stage (total 540 cases) or Fuhrman grade (total 486 cases) (Supplementary Table 2). To analyze RKIP expression, we divided the cases with H-Score ≥ 260 to the strong expression group and the cases with H-Score < 260 to the weak expression group. This system has been widely used to analyze TMA [41,42,34]. In organ-confined ccRCCs, the number of carcinomas in the strong expression group was clearly decreased from T2N0M0 tumors to T3N0M0 carcinomas as well as from Fuhrman grade 1 tumors to Fuhrman grade 2 carcinomas (Fig. 3B, C). The levels of RKIP expression based on the H-Scores were also significantly decreased from NKT to ccRCC, from T2N0M0 to T3N0M0, and from Fuhrman grade 1 to Fuhrman grade 2 (Fig. 3D, E). While we could not establish a significant RKIP reduction from organ-confined ccRCC without metastasis to local carcinomas with metastasis, which was likely attributable to our limited cases of the latter group, a trend of RKIP reduction was observed (Fig. 3F). Within the 15 cases of local carcinoma with metastasis, tissues from the metastasized organ (adrenal gland, bone, lymph node, lung, thyroid, intestine, and spleen) were available for 8 cases; the RKIP levels were significantly lower in metastasized ccRCC compared to the local counterparts (Fig. 3F, G).

To further examine RKIP expression in metastasized ccRCC, we obtained 7 clinically confirmed metastasized ccRCCs from hospitals in Hamilton, including one tissue each from lymph node, back subcutaneous, colon, and lung chest wall, and three lung metastases (data not shown). At comparable staining conditions, RKIP expression was dramatically reduced in metastasized ccRCC compared to the non-tumor kidney tissues (data not shown). Taken together, in the total of 15 cases of metastasized ccRCC obtained from different patient cohorts RKIP expression was drastically reduced compared to NTK tissues.

We also determined whether RKIP levels associate with patient survival. In the patient population included in the TMAs (Supplementary Table 2), there were 25 patients with follow-up information. We noticed that organ-confined ccRCCs without metastasis, with metastasis, and metastasized ccRCC have an average H-Score of RKIP at 220, 204, and 181, respectively (Fig. 3F). These patients were thus analyzed based on H-Score > 204 or ≤ 204. Among 13 patients with RKIP > 204, 23% (3/13) died of ccRCC; in 12 patients with RKIP ≤ 204, 50% (6/12) died of cancer. To increase patient numbers, a new TMA was analyzed, which contained 14 RKIP-positive and 32 RKIP-negative ccRCC (Supplementary Table 3); 3/14 (21.4%) RKIP-positive and 12/32 (37.5%) RKIP-negative ccRCC patients died from the disease (Supplementary Table 3). Kaplan-Meier survival analysis indicated that patients with RKIP-negative ccRCC were likely to have poorer survival than those with RKIP-positive ccRCC (Fig. 3H). Similar results were also obtained when the *RKIP* transcription data in Oncomine™ were analyzed [43] (data not shown). However, in all these analyses, the differences were not statistically significant (Fig. 3H; data not shown). This is likely due to the limited number of patients available with followed-up information and the lack of sufficient number of patients with RKIP-positive ccRCC, as a result of a common RKIP reduction, adds an additional challenge.

### Transcription regulation contributes to RKIP reduction in ccRCC

**Figure 4:**
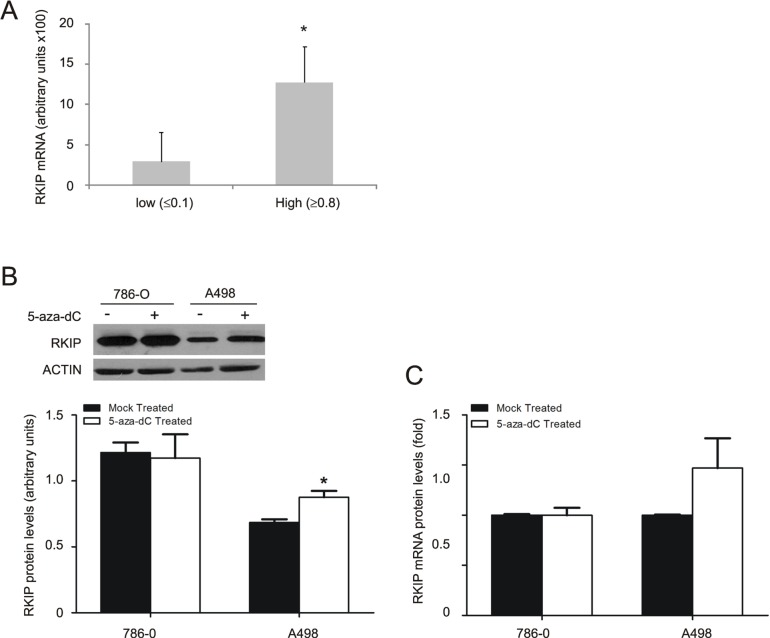
Regulation of RKIP transcription contributes to decease of RKIP in ccRCC (**A**) Real-time PCR analysis of the RKIP transcripts in ccRCCs that express low or high levels of RKIP protein (see Supplementary Table 1 for details). Experiments were repeated three times. The relative abundance of RKIP mRNA was in reference to actin and means ± SD (standard derivation) are graphed. * *p* < 0.05 in comparison to the low group (2-tailed student t-test). (**B and C**) 786-0 and A498 cells were either mock-treated or treated with 5-aza-dC (2 μM) for 48 hours, followed by examination of the RKIP protein by western blot (**B**) and the RKIP transcript by real time PCR (C). Experiments were repeated in triplicate and means ± SE are graphed. * *p* < 0.05 in comparison to the mock-treatment (2-tailed student t-test).

To investigate the mechanisms responsible for the observed RKIP reduction, we examined the levels of RKIP mRNA using real time PCR. While ANK tissues consist of multiple cell types derived from glomeruli and distal tubules, RKIP is predominantly expressed in the proximal tubule epithelial cells (Fig. 1C). As there is no bona fide markers available specific for the proximal tubular epithelial cells, in which ccRCC is originated, it is impossible to compare the abundance of RKIP mRNA in the proximal tubular epithelial cells with that in ccRCC. However, we noticed that the RKIP protein levels in ccRCC vary significantly (Supplementary Table 1). Based on the ratios of RKIP protein (ccRCC/ANK), we first grouped ccRCCs into two levels, low (≤ 0.1) and high (≥0.8) (Supplementary Table 1). After normalization to actin, semi-quantitative real time PCR (qRT-PCR) demonstrated that RKIP mRNA levels followed the same order in the two groups (Fig. 4A). The ccRCC tumors were confirmed by the elevation of VEGF-A compared to ANK (data not shown). Collectively, these results support the notion that inhibition of RKIP transcription plays a role in its reduction in ccRCC.

To consolidate this concept, we demonstrated decreases in the RKIP protein in A498 and 786-0 ccRCC lines in comparison to human kidney proximal tubular epithelial HK2 cells (Supplementary Fig. 1). Promoter methylation has been shown to repress RKIP transcription [44]. To examine whether methylation is involved in RKIP reduction, A498 and 786-0 cells were treated with 5-aza-2'-deoxycytidine (5-aza-dC, a widely used DNA methyltransferase inhibitor) at 2 μM for 48 hours. 5-aza-dC increased RKIP at both mRNA and protein levels only in A498 cells (Fig. 4B, C). This is consistent with the observation that in comparison to HK2 cells RKIP is significantly reduced in A498 cells (Supplementary Fig. 1). Collectively, the above results reveal that transcriptional regulation contributes in part to the decrease of RKIP in ccRCC.

### RKIP does not affect ccRCC proliferation and the formation of xenograft tumors

The most well characterized function of RKIP is the inhibition of Raf-1-mediated ERK activation. To determine the relationship between RKIP and ERK activation, western blot analysis was performed on 50 cases of ccRCC; significant changes in ERK protein and in ERK activation between ccRCC and ANK could not be demonstrated (data not shown). This is consistent with the ERK activation levels being not reversely correlated with the RKIP protein abundance in A498, 786-0, and HK2 cells (Supplementary Fig. 1).

**Figure 5:**
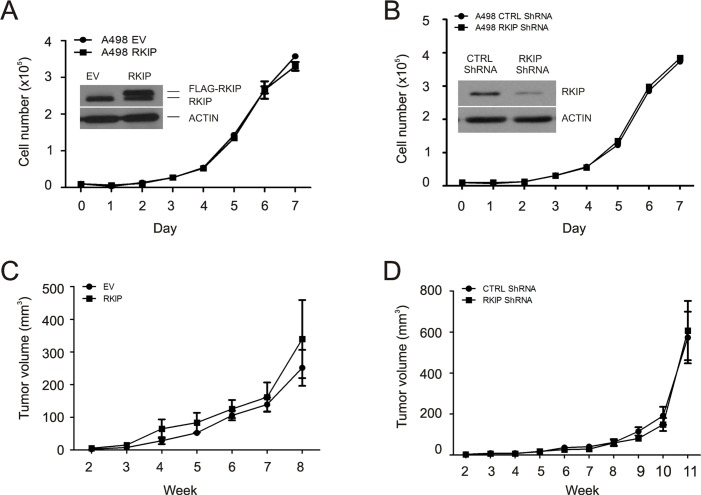
Modulation of RKIP does not affect A498 cell proliferation and neither the cell's ability to form xenograft tumors in NOD/SCID mice (**A and B**) A498 cell lines were constructed to express an empty vector (EV), FLAG-tagged RKIP, control (Ctrl) shRNA or RKIP shRNA. Ectopic expression and knockdown of RKIP were confirmed (insets). Cell's proliferation ability was then determined. (**C and D**) A498 EV, RKIP, Ctrl shRNA, and RKIP shRNA cells (3×10^6^) were implanted into NOD/SCID mice (5 mice per cell line). Tumor volumes were measured weekly. Xenograft tumors were formed in all five mice. Mean volumes ± SE were plotted.

We subsequently examined the impact of RKIP on ccRCC cell proliferation. Ectopic expression of FLAG-tagged RKIP in A498 and 786-0 cells did not affect their proliferation (Fig. 5A, Supplementary Fig. 2A). Knockdown of endogenous RKIP in either line was also without effects (Fig. 5B, Supplementary Fig. 2B). Consistent with ERK activity promoting cell proliferation, these results are in line with the observations that RKIP expression did not reversely correlate with ERK activation in primary ccRCC (data not shown), A498 and 786-0 cells (Supplementary Fig. 1).

We further determined whether RKIP inhibits ccRCC tumorigenesis. A498 EV (empty vector), A498 RKIP, A498 Ctrl shRNA, or A498 RKIP shRNA cells were subcutaneously implanted into NOD/SCID mice (5 mice/cell line). Tumor incidence for all lines was 5 for 5. The kinetics of xenograft tumor formation did not differ between EV and RKIP cells as well as between Ctrl shRNA and RKIP shRNA cells (Fig. 5C, D). Stable modulations of RKIP expression were demonstrated in A498 cells (Fig. 5A, B, see the insets) and in xenograft tumors (Supplementary Fig. 3). Collectively, we conclude that RKIP does not play a major role in inhibiting ERK activation, cell proliferation, and the formation of xenograft tumors in ccRCC cells. These observations are consistent with the reports that overexpression of RKIP did not affect the proliferation of breast and prostate cancer cells *in vitro* and their ability to form xenograft tumors *in vivo* [26,41].

### RKIP reduces ccRCC cell invasion

**Figure 6:**
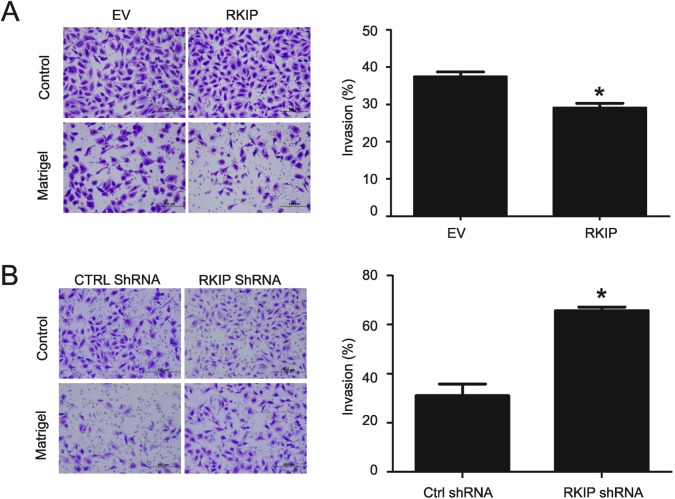

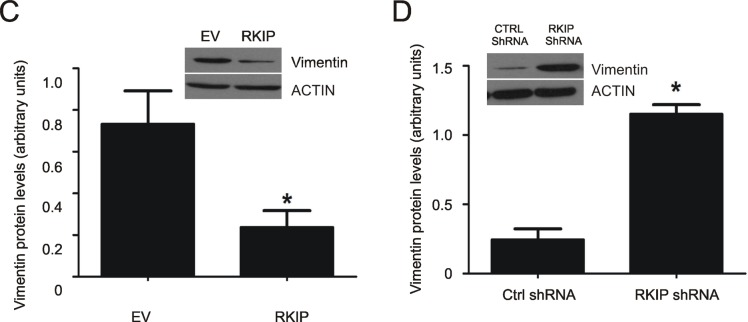
Modulations of RKIP affect A498 ccRCC cell invasion (**A and B**) The indicated A498 cell lines were assayed for their invasion ability using either a control membrane or 8 μM matrigel membranes. Typical image of cells that have passed through either membrane are shown (left panels). Quantification of three independent repeats (means ± SE) is also graphed (right panels). * *p* < 0.01 in comparison to the respective controls (2-tailed student t-test). (**C and D**) A498 EV, RKIP, Ctrl shRNA, and shRKIP cells were examined for the expression of vimentin and actin by western blot (see the insets for typical results). Vimentin in individual cell lines were normalized to the respective actin. Experiments were repeated in triplicate. Means ± SE are graphed. * *p* < 0.05 in comparison to either A498 EV or Ctrl shRNA cells (2-tailed student t-test).

RKIP has been shown to inhibit breast and prostate cancer metastasis [26,41]. Since the invasion ability of a cell is closely related to cancer's metastatic potential, we have determined the impact of RKIP on ccRCC cell invasion. Overexpression of RKIP in A498 and 786-0 cell lines reduced their invasion ability in comparison to the EV cells (Fig. 6A, Supplementary Fig. 4A). Conversely, knockdown of RKIP in both lines robustly enhanced their invasion capacity (Fig. 6B, Supplementary Fig. 4B).

EMT is an essential process of metastasis [23,24]; RKIP inhibits EMT in breast and prostate cancers [26,41]. We thus examined the potential impact of RKIP on EMT in ccRCC cells. As most (95%) of primary ccRCCs do not express E-cadherin [42] and neither A498 and 786-0 cells (data not shown), we determined the impact of RKIP on N-cadherin and vimentin, two-well recognized EMT markers. Ectopic expression and knockdown of RKIP modestly but significantly inhibited and enhanced N-cadherin expression in A498 cells, respectively (data not shown). Meanwhile, overexpression and knockdown of RKIP significantly downregulated and upregulated Vimentin expression in A498 cells, respectively (Fig. 6C, D). Collectively, these observations support the notion that RKIP reduces ccRCC invasion at least in part via inhibiting EMT.

## DISCUSSION

Clear cell renal cell carcinoma is commonly associated with upregulation/activation of VEGF (vascular endothelial growth factor), PDGF-β (platelet-derived growth factor), and TGF-α [45,46]. These growth factors activate a major oncogenic event - the Raf pathway. Consistent with the pathway playing an important role in ccRCC evolution, we provide evidence that reduction of RKIP might facilitate ccRCC tumorigenesis.

Our observed reduction of RKIP in 80% of more than 600 ccRCC cases examined strongly suggests RKIP being a candidate gatekeeper for ccRCC. As VHL is reduced or lost in 75% of ccRCC cases, it can be predicted that at least 60% of ccRCCs will have both proteins reduced. This is in accordance with our observations in which 78% (39/50) of ccRCC have both incidences (data not shown). It will be interesting to examine how these two events together may impact ccRCC tumorigenesis. RKIP may not only be a gatekeeper for ccRCC, as reduction of RKIP has been observed in 50% of prostate cancers [47]. Regardless whether RKIP functions as a gatekeeper for ccRCC, prostate cancer, and other human cancers, it seems that reduction of RKIP needs other oncogenic signals for malignancy. This is because RKIP deficient mice do not develop tumors [48].

While RKIP is dominantly reduced in majority of ccRCC, modulations of RKIP expression did not affect A498 cell's ability to produce xenograft tumors in NOD/SCID mice. Because A498 cells are cancerous cells, in which many changes have occurred, the above observations may not truly reflect RKIP's ability in suppressing the formation of primary ccRCC. Better animal models i.e. knockout and transgenic mice will be needed to conclusively address this issue. However, the observation that modulations of RKIP accordingly affected the invasion of A498 and 786-0 cells suggests that RKIP inhibits ccRCC metastasis. Similar observations were reported in prostate cancer, in which RKIP reduction affected the cell's metastatic but not xenograft tumor forming ability [26,47]. This is in line with RKIP being an independent prognostic marker for patients with Dukes' B colorectal cancer [49].

Although RKIP was best studied for its inhibition of Raf-initiated activation of MEK [50], an upstream kinase of ERK, it does not appear that RKIP suppresses ccRCC tumorigenesis mainly via inhibiting the Raf-MEK-ERK pathway (Supplementary Fig. 1, data not shown). This is consist with our observation that phosphorylation on S153 of RKIP was not changed in ccRCC compared to that in the ANK tissues (data not shown) and this phosphorylation inactivates RKIP's ability to prevent Raf from activating MEK [18]. On the other hand, evidence supports that RKIP inhibits ccRCC tumorigenesis and/or metastasis via inhibiting EMT, based the observations that modulations of RKIP accordingly affected A498 invasion along with changes in vimentin expression (Fig. 6). However, it remains unclear whether RKIP reduces vimentin expression at mRNA or protein levels. While 5-aza-dC treatment elevated RKIP expression in A498 cells (Fig. 4B, C), the treatment did not increase vimentin mRNA abundance (data not shown).

Although whether RKIP primarily inhibits ccRCC tumorigenesis and/or metastasis and the underlying mechanisms need further investigations, our observations that the RKIP protein is surprisingly reduced in 80% of ccRCC strongly suggests a critical role of RKIP in preventing ccRCC tumorigenesis. Our research is consistent with a recent report showing decreases of RKIP in 42.2% of RCC and the association of RKIP reduction with RCC progression [51]. What are the mechanisms responsible for RKIP reduction in ccRCC is also worthy of future investigation.

## MATERIALS AND METHODS

### Tissue Collection

Kidney cancer and the adjacent non-tumor (ANK) tissues were collected at St. Joseph's Hospital in Hamilton, Ontario, Canada with consent from patients and approval of the Research Ethics Board.

### Modulation of gene expression in ccRCC cells

Stable expression of RKIP in A498 and 786-0 ccRCC cells was achieved using retrovirus as we have previously described [52]. Knockdown of RKIP in A498 and 786-0 cells was carried out using a pRIH retroviral-based shRNA vector according to our published conditions [52]. The RKIP targeting sequence was 5'– GTGGGATGGTCTTGATTCA –3'. Both A498 and 786-0 ccRCC lines are VHL deficient [53]

### Cell proliferation and invasion assay

Cells were seeded at a density of 10^4^ per well in 6-well plates and cell numbers were determined daily for seven days. Triplicate wells were counted each day.

Invasion assays were performed using an 8 μm pore size matrigel membrane as previously described [54]. Briefly, insert chambers were placed into a 24-well plate (BD Biosciences). Serum containing medium was placed in the bottom chamber and cells suspended in serum-free medium were added to the top chamber. Cells that passed through control or matrigel membranes were stained with crystal violet. Percentage of invasive cells was calculated by dividing the number of cells passing through the 8 μm pore size matrigel membrane by the number of cells migrating through the control membrane and multiplying by 100.

### Western blot analysis, immunohistochemistry (IHC), and real time PCR

Cell lysates were prepared and western blot was performed using anti-RKIP (1:500, Santa Cruz), anti-ERK (1:1000, Cell Signaling), anti-phosphoERK (1:1000, Cell Signaling), anti-FLAG (1:1000, Sigma), anti-N-Cadherin (1:1000, Sigma), anti-Vimentin (1:500, Santa Cruz), and anti-actin (1:1000, Santa Cruz).

IHC was executed using a set of tissue microarray (TMA) slides (KD806, KD951, KD2085, KD2088, and KD6161) from US Biomax (Rockville, MD) and IMH-313 from Imgenex (San Diego, CA). Primary anti-RKIP antibody (1:500, Santa Cruz) was incubated with the sections overnight at 4°C. Biotinylated secondary IgG and Vector ABC reagent (Vector Laboratories) were subsequently added according to the manufacturer's instructions. TMA slides were scanned using ScanScope and analyzed using ImageScope software (Aperio); scores obtained were converted to H-Score using the formula: H-Score = (% weak × 1 + % medium × 2 + % strong × 3 +1) × 100 [55,56,52]. H-Scores ≥ 260 and < 260 were considered strong and weak staining, respectively [55,56,52]. Each cancerous tissue core was in duplicate. All spots (stained cores) were also manually examined. Tissue cores were excluded from analysis if the tissue was scratched or missing majority of the sample.

Total RNA was isolated using TRIZOL (Invitrogen). Reverse transcription was carried out using superscript III (Invitrogen) according to manufacturer's instruction. Real time PCR primers used for RKIP, VEGF, and actin are listed in Supplementary Table 4. Real-time PCR was performed using the ABI 7500 Fast Real-Time PCR System (Applied Biosystems) in the presence of SYBR-green according to the manufacturer's instructions (Applied Biosystems). Gene expression was measured relative to actin expression using the following formula for the relative transcript abundance (RTA): RTA = 10,000/2^(CTgene-CTactin)^ [57].

### Generation of xenograft tumors

A498 cells (3×10^6^) overexpressing an empty vector (EV), RKIP (RKIP), short hairpin control (Ctrl ShRNA) or RKIP ShRNA were resuspended into a MEM/Matrigel mixture (1:1 volume) and subcutaneously implanted into the flank of NOD/SCID mice (The Jackson Laboratory) with each group containing 5 mice. Tumor volume was calculated according to the formula *L* × *W*^2^ × 0.52, where *L* and *W* are the longest and shortest diameters respectively [58]. All animal work was performed according to protocols approved by the McMaster University Animal Research Ethics.

### Statistical analysis

All data are presented as mean ± standard error (SE). Statistical analysis was carried out using the statistical program SPSS Statistics 17.0 for Windows. Two-tailed Student's T-Test was used. Pearson's correlation was used to test relationships between RKIP protein expression and tumor stage and tumor grade progression. One way analysis of variance (ANOVA) was performed to evaluate effect of tumor stage, tumor grade and metastasis on H-Score. A *p*-value <0.05 was considered statistically significant.

### SUPPLEMENTAL FIGURES AND TABLES


