# Supplementary material for: Common reduction of the Raf kinase inhibitory protein in clear cell renal cell carcinoma
Source: Oncotarget. 2014 Apr 24;5(17):7406–19. doi: 10.18632/oncotarget.1558 (PMC4202132; doi:10.18632/oncotarget.1558)
Supplement: Supplementary file 1 [file oncotarget-05-7406-s001.pdf]

SUPPLEMENTARY FIGURES AND TABLES

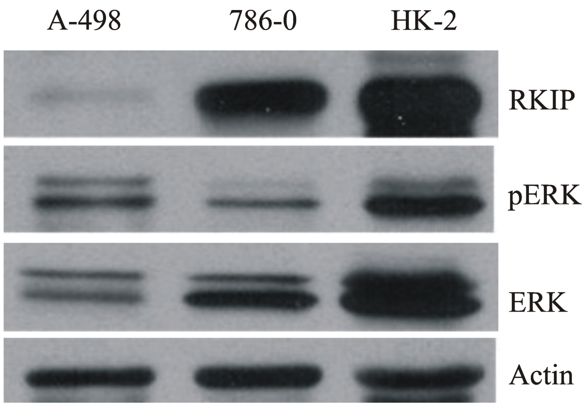

Supplementary Figure 1. Reduction of RKIP in ccRCC cell lines. Human proximal tubular epithelial cells (HK-2) and ccRCC cell lines (786-0 and A-498) were examined by western blot for the expression of RKIP, pERK (phosphorylated/active ERK), ERK, and actin. Experiments were repeated twice. Typical images from a single repeat are shown.

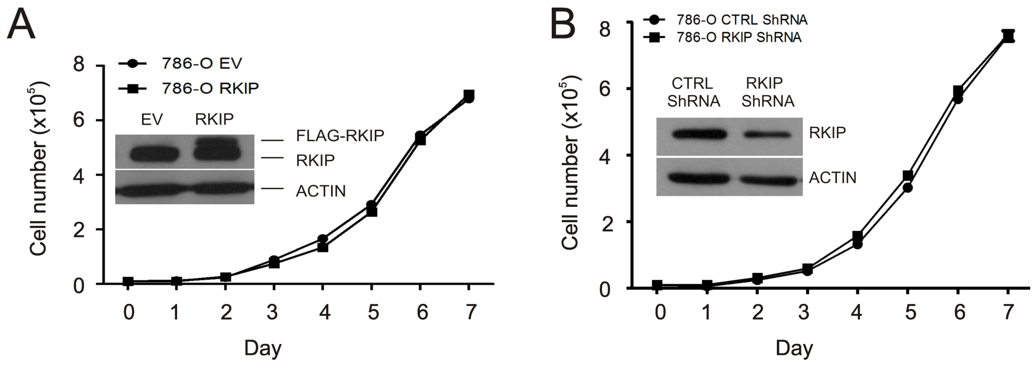

Supplementary Figure 2. Modulation of RKIP does not affect 786-0 cell proliferation. (A) 786-0 cell lines were constructed to express an empty vector (EV) or FLAG-tagged RKIP (inset). Cell's proliferation ability was then determined. (B) 786-0 cells were stably infected with a control (CTRL) shRNA or RKIP shRNA retrovirus. Knockdown of RKIP was confirmed by western blot (inset). Cell's proliferation potential was subsequently examined (see Materials and Methods for details methodology).

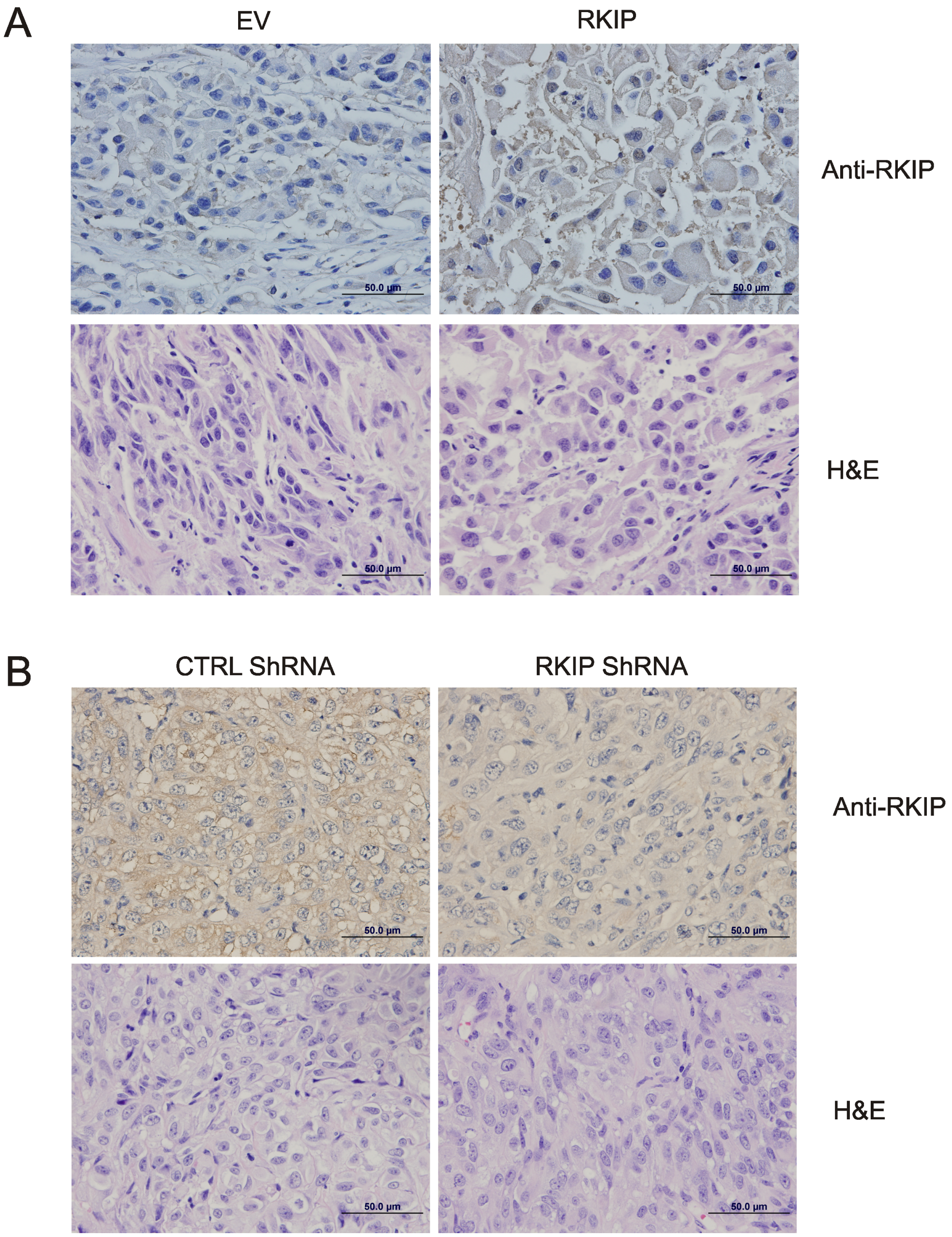

Supplementary Figure 3. Confirmation of RKIP status in xenograft tumors. A498 EV, A498 RKIP, A498 Ctrl shRNA, and A498 RKIP shRNA cell ($3x10^6$) cells were implanted into NOD/SCID mice (5 mice per cell line). Xenograft tumors generated from EV and RKIP (A) as well as Ctrl shRNA and RKIP shRNA cells (B) were examined for RKIP by IHC. H&E staining was also performed.

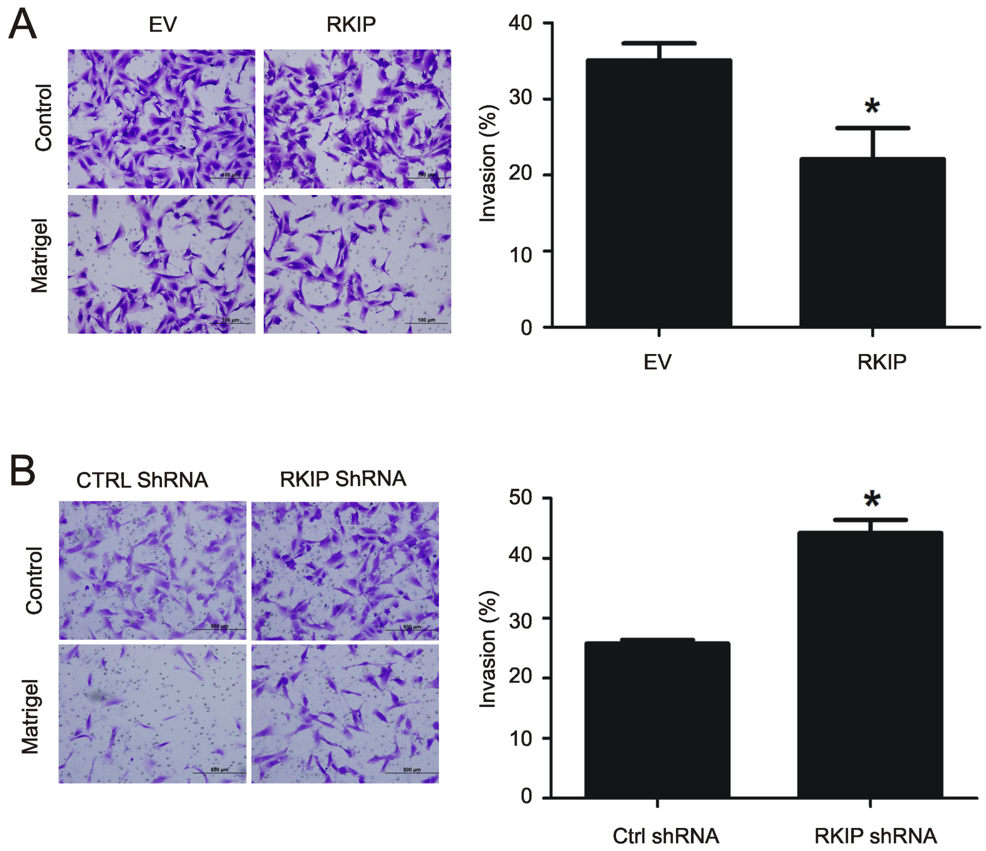

Supplementary Figure 4. Modulations of RKIP affect 786-0 ccRCC cell invasions. (A, B) the indicated 786-0 cell lines were assayed for their invasion capacity using either a control or 8 μM matrigel membrane. Typical image of cells that have passed through either the control or matrigel membrane are shown (left panels). Quantifications of three independent repeats (means ± SE) are also graphed (right panels). * $p < 0.01$ in comparison to the respective controls (2-tailed student t-test).

Supplementary Table 1: Patient's clinical information and their associated RKIP expression

Patients	Pathology	Age	Gender	Fuhrman	Metastas	Tumor size	ANK[1]	ccRCC[2]	C/N[3]
1	ccRCC	54	M	3	No	5.5 cm	0.127717	0.07581	0.593
2[#]	ccRCC	65	M	4	Yes -lung	8 cm	0.674501	0.070167	0.104
3	ccRCC	54	F	4	Yes		1.741366	0.678187	0.389
4	ccRCC	54	M	1	No	3.6 cm	0.446428	0.064784	0.145
5[#]	ccRCC	48	M	3	No	6 cm	0.705356	0.02246	0.031
6	ccRCC	75	F	3	No	11 cm	0.169396	0.043553	0.257
7	ccRCC	38	M	2	No	2.9 cm	1.45	0.130922	0.090
8[#]	ccRCC	63	M	2	No	11.5 cm	2.370187	0.193881	0.081
9	ccRCC	77	M	2	No	6.5 cm	0.960128	0.151572	0.157
10	ccRCC	63	M	3	No	4.5 cm	1.30682	0.287429	0.219
11	ccRCC	70	F	1	No	4 cm	1.456749	0.334355	0.229
12	ccRCC	63	M	3	No	3.5 cm	1.382522	0.313858	0.227
13	ccRCC	58	M	2	No	2 cm	1.445312	0.546285	0.377
14[¥]	ccRCC	58	M	2	No	5.4 cm	0.486487	0.599981	1.233
15	ccRCC	51	M	1	No	4.5 cm	1.806974	0.087541	0.048
16[¥]	ccRCC	54	F	1	No	6.2 cm	0.63797	0.693541	1.087
17	ccRCC	77	F	2	No	5.5 cm	1.269767	0.72066	0.567
18	ccRCC	48	M	3	No	13.5 cm	1.270234	0.475082	0.374
19	ccRCC	45	M	3	No	4.5 cm	1.043326	0.150719	0.144
20	ccRCC	41	F	1	No	6.2 cm	2.168121	0.711055	0.327
21	ccRCC	68	M	3	No	6 cm	1.740729	0.916528	0.526
22	ccRCC	57	M	3	Yes	9 cm	0.564783	0.41376	0.732
23	ccRCC	55	M	4	No	13 cm	1.572677	0.519176	0.330
24[#]	ccRCC	76	M	2	Yes -bone	6.2 cm	1.596312	0.046612	0.029
25	ccRCC	68	F	2	No	3.5 cm	2.689019	0.560247	0.208
26	ccRCC	59	M	2	No	5.5 cm	0.818924	0.279611	0.341
27	ccRCC	49	F	2	No	5 cm	1.193792	0.42416	0.355
28[#]	ccRCC	43	M	2	No	3 cm	1.825573	0.092531	0.050
29	ccRCC	68	M	3	No	4.5 cm	0.458977	0.179648	0.391
30	ccRCC	50	M	1	No	1.7 cm	0.446257	0.062582	0.140
31	ccRCC	83	F	2	No	8 cm	0.699453	0.286779	0.410
32	ccRCC						0.767934	0.192831	0.251
33	ccRCC	56	F	3	No	3 cm	0.364325	0.135054	0.370
34	ccRCC	71	M	3	No	12.5 cm	0.175649	0.08613	0.490
35[¥]	ccRCC	67	F	2	No	3.2 cm	0.1326	0.107878	0.813
36	ccRCC	62	M	3	No	3 cm	0.153308	0.078777	0.513

(*Continued*)

Patients	Pathology	Age	Gender	Fuhrman	Metastas	Tumor size	ANK[1]	ccRCC[2]	C/N[3]
37	ccRCC		F	2	No	2 cm	0.40706	0.059358	0.145
38	ccRCC	91	F	2	No	2.7 cm	0.457912	0.14268	0.311
39	ccRCC	72	F	2	No	3.5 cm	0.496548	0.305436	0.615
40	ccRCC	48	F	2	No	1 cm	0.720965	0.077126	0.106
41	ccRCC	62	M	3	No	3.5 cm	0.138263	0.051452	0.372
42	ccRCC	75	M	2	No	6 cm	0.032308	0.010309	0.319
43	ccRCC	74	M	2	No	3.5cm	0.427803	0.05628	0.131
44	ccRCC	45	F	1	No	2.5 cm	0.348807	0.096062	0.275
45	ccRCC	48	M	1		5 cm	0.274534	0.07416	0.270
46	ccRCC	62	M	2	No		0.710442	0.022004	0.030
47	ccRCC	53	F	1	No	5.6 cm	0.574011	0.04837	0.084
48[¥]	ccRCC	61	M	1	No	5.5 cm	0.229504	0.318556	1.388
49[¥]	ccRCC	46	M	2	No	3.5 cm	0.032964	0.149353	4.53
50[¥]	ccRCC	49	F	1	No	4 cm	0.052059	0.828449	15.914

[#]: The ratios (ccRCC/ANK) of RKIP expression ≤ 0.1 (the low level group)
[¥]: The ratios (ccRCC/ANK) of RKIP expression = 0.8-1.2 (the high level group)
[1]RKIP in individual ANK tissues was normalized to actin
[2]RKIP in individual ccRCC tissues was normalized to actin
[3]Ratios between ANK (N) and ccRCC (C)

Supplementary Table 2: Patient composition for the set of TMA slides[1]

Tumor Stage[2]	Number of cases	Tumor Grade[3]	Number of cases[4]
T1N0M0	254	1	277
T2N0M0	183	2	194
T3N0M0	97	3	15
T4N0M0	6		
Total	540	Total	486

[1]TMA slides from US Biomax: KD806, KD951, KD2085, KD2088 and KD6161
[2]TMN stage: T – tumor size, N – lymph node metastasis, M – long distance metastasis
[3]Fuhrman Grade
[4]Some (54) ccRCC casers did not have tumor grade information

Supplementary Table 3: Pathological information for patients included in TMA IMH-313

#	Age	Sex	Diagnosis	Type	Size (cm)	Capsule	Vein	T	N	M	Follow Up (Months)	Follow Up Result	Cause of Death	RKIP
**1**	52	F	RCC	G	12	+	−	T2b	NX	M0	119	Alive	.	+
**2**	60	F	RCC	CC	6	+	+	T3a	N0	M1	118	Alive	.	−
**3**	42	M	RCC	CC	9	+	+	T3a	NX	M0	65	Dead	Cancer	−
**4**	59	M	RCC	CC	7.5	+	−	T2a	NX	M0	116	Alive	.	−
**5**	67	F	RCC	CC + G	8	+	−	T2a	NX	M0	116	Alive	.	−
**6**	65	M	RCC	CC + G	11	+	−	T2b	NX	M0	115	Alive	.	+
**7**	59	M	RCC	CC + G	7	+	+	T1b	NX	M0	4	Dead	Cancer	−
**8**	56	M	RCC	C + Pseudo	7	+	−	T1b	NX	M1	43	Dead	Cancer	−
**9**	42	F	RCC	CC	10	−	+	T3a	NX	M0	116	Alive	.	−
**10**	50	M	RCC	CC	3.5	−	−	T1a	N0	M0	113	Alive	.	+
**11**	52	M	RCC	CC	4	−	−	T1a	N0	M0	112	Alive	.	−
**12**	68	F	RCC	CC	6	+	+	T3a	N0	M0	112	Alive	.	−
**13**	60	F	RCC	CC	5	−	+	T3a	N0	M0	32	Dead	Cancer	−
**14**	69	F	RCC	CC	7.5	+	−	T2a	NX	M	111	Alive	.	+
**15**	54	M	RCC	CC	7	+	−	T1b	NX	M0	110	Alive	.	−
**16**	43	F	RCC	CC	4	+	−	T1a	N0	M0	110	Alive	.	−
**17**	53	M	RCC	CC	5.5	−	−	T3a	NX	M1	110	Alive	.	−
**18**		M	RCC	CC	3.5	−	+	T3a	N0	M0	109	Alive	.	+
**19**			RCC	CD	4	+	−	T1a	NX	M0	108	Alive	.	−
**20**	40	M	RCC	CC	2.2	−	−	T1a	NX	M0	108	Alive	.	−
**21**	52	M	RCC	CC	6	+	−	T1b	NX	M0	107	Alive	.	−
**22**	63	F	RCC	CC	7.5	+	−	T2a	NX	M0	85	Dead	Cancer	−
**23**	64	M	RCC	CC	5.4	+	−	T1b	NX	M0	59	Dead	Chronic Renal Failure	−
**24**	37	M	RCC	CD	10.5	−	−	T2b	N0	M0	15	Dead	Cancer	−
**25**	65	M	RCC	P	5	−	−	T1b	NX	M0	106	Alive	.	−
**26**	74	M	RCC	CC	17	−	+	T3a	N0	M0	90	Dead	Cancer	−
**27**	67	M	RCC	CC	9	+	+	T4	NX	M0	22	Dead	Cancer	−
**28**	50	M	RCC		9	−	−	T2a	N0	M0	105	Alive	.	−
**29**	74	M	RCC	CC	17	−	+	T3a	N0	M0	90	Dead	Cancer	+
**30**	62	M	RCC	CC + G	7	+	+	T3a	NX	M0	105	Alive	.	−
**31**	64	M	RCC	P	4	−	−	T1a	NX	M0	105	Alive	.	+
**32**	45	M	RCC	CC	6.5	−	−	T1b	N0	M0	105	Alive	.	+
**33**	62	M	RCC		10	+	−	T3a	N1	M0	30	Dead	Cancer	−

(*Continued*)

#	Age	Sex	Diagnosis	Type	Size (cm)	Capsule	Vein	T	N	M	Follow Up (Months)	Follow Up Result	Cause of Death	RKIP
**34**	53	M	RCC	CC	4.5	+	–	T1b	NX	M0	104	Alive	.	–
**35**	64	M	RCC	CC	6	–	–	T1b	NX	M0	104	Alive	.	–
**36**	72	M	RCC	CC	1.5	–	–	T1a	NX	M0	104	Alive	.	+
**37**	62	F	RCC	CC	11	–	–	T3a	NX	M0	103	Alive	.	–
**38**	56	M	RCC	CC	4	+	+	T3a	NX	M0	31	Dead	Cancer	+
**39**	58	M	RCC		8.5	–	–	T2a	NX	M0	101	Alive	.	–
**40**	57	M	RCC		10	+	–	T3b	NX	M0	101	Alive	.	–
41	*64*	*F*	*Transitional cell carcinoma*								*0*	*Dead*	*Cancer*	+
**42**	43	F	RCC	CC	6	–	–	T1b	N0	M0	100	Alive	.	–
**43**	52	M	RCC	G	7	–	–	T1b	NX	M0	100	Alive	.	+
**44**	64	M	RCC	G	10	–	+	T3a	NX	M0	4	Dead	Cancer	–
**45**	57	M	RCC	CC	4.5	+	–	T1b	NX	M0	99	Alive	.	+
**46**	59	M	RCC	CC	8	+	–	T2a	NX	M0	38	Dead	Cancer	–
**47**	47	M	RCC	CC	4	+	–	T1a	NX	M0	98	Alive	.	+
**48**	77	M	RCC	CC	2.5	+	–	T1a	NX	M0	18	Dead	Cancer	–
49	*47*	*F*	*RCC*	*CC + G*	*8*	+	–	*T2a*	*NX*	*M0*	*22*	*Dead*	*Breast Cancer*	+
**50**	55	F	RCC	CC	3	+	–	T3a	NX	M0	97	Alive	.	+
**51**			Normal	#4										+
**52**			Normal	#16										+
**53**			Normal	#20										+
**54**			Normal	#21										+
**55**			Normal	#30										+
**56**			Normal	#36										+
**57**			Normal	#42										+
**58**			Normal	#48										+
**59**			Normal	#50										+

The TMA IMH-313 was from Imgenex; San Diego, CA. Cancer types included are; G: Granular Cell type, CC: Clear Cell type, Pseudo: Pseudosarcomatous type, CD: Collecting Duct type, P: Papillary type. Italicized samples (Patient 41 and 49) were not included in the analysis due to deaths unrelated to RCC. RKIP+ and RKIP- was defined by H-Score >204 and ≤ 204, respectively.

Supplementary Table 4: Real-time PCR primers

Gene	Primer Sequence	Reference[$]
RKIP (Human)	forward – 5'-AGACCCACCAGCATTTCGTG-3'<br>reverse – 5'-GCTGATGTCATTGCCCTTCA-3'	Zaravino *et al,* 2008
VEGF (Human)	forward – 5'-AAGGAGGAGGGCAGAATCAT-3'<br>reverse – 5'-CCAGGCCCTCGTCATTG-3'	Thijssen et al, 2004
β-Actin (Human)	forward – 5'-CCCTGAAGTACCCCATCGAG-3'<br>reverse – 5'-CAGATTTTCTCCATGTCGTCCC-3'	Sloan et al, 2009

[$]Real time primers were reported in:

Zaravinos A, Bizakis J, Spandidoe DA. RKIP and BRAF aberrations in human nasal polyps and the adjacent turbinate mucosae. *Cancer Letters* 2008; **264:** 288–298.

ThijssenV, Brandwijk R, Dings R *et al.* Angiogenesis gene expression profiling in xenograft models to study cellular interactions. *Experimental Cell Researc*h 2004; **299:** 286–293.

Sloan KA, Marquez HA, Li J *et al.* Increased PEA3/E1AF and decreased Net/Elk-3, both ETS proteins, characterize human NSCLC progression and regulate caveolin-1 transcription in Calu-1 and NCI-H23 NSCLC cell lines. *Carcinogenesis* 2009; **30:** 1433–1442.
